# Analysis of the Influence of Crack Position and Orientation on the Stability of a Flat Al7075-T651 Plate Using the Finite Element Method and the Failure Assessment Diagram

**DOI:** 10.3390/ma19122555

**Published:** 2026-06-12

**Authors:** Liviu Daniel Pîrvulescu, Dorin Bordeasu, Florin Dragan

**Affiliations:** 1 Department of Mechanics and Strength of Materials, Bulevardul Mihai Viteazu 1, No. 1, 300222 Timisoara, Romania; liviu-daniel.pirvulescu@upt.ro; 2 Department of Automation and Applied Informatics, Politehnica University of Timisoara, Vasile Parvan No. 2, 300223 Timisoara, Romania; florin.dragan@upt.ro

**Keywords:** Al7075-T651, aluminum alloy, failure assessment diagram, finite element method, fracture mechanics, stress intensity factor, crack position, crack orientation, crack size, material analysis

## Abstract

Aluminum is undoubtedly a key material in modern industry. Flat plates made of aluminum alloys are widely used in construction, aeronautics, automotive, and others. The current paper presents an analysis of the behavior of a thin plate made of Al7075-T651 aluminum alloy, subjected to a uniaxial stress, and clamped at one end. The results of the numerical simulation with FRANC2D software have been used for accurate determination of the stress intensity factors (K_I_, K_II_) and being validated for the simple cases using analytical calculations. The Failure Assessment Diagram (FAD) based on the toughness ratio Kr and the load ratio Lr has been used to evaluate the structural integrity of cracked components based on the load, its position, crack size, and the fracture properties of the material. The FAD analysis results highlight the significant influence of crack position on the values of the K factor. The edge and inclined cracks lead to increases in stress intensity factors and to the occurrence of mixed-mode loading conditions. The study demonstrates the effectiveness and usefulness of the proposed methodology in the analysis of structures with discontinuities and emphasizes the importance of crack positioning in assessing the safety of engineering components.

## 1. Introduction

Structural integrity is a critical aspect of mechanical engineering, particularly in fields such as aerospace, transportation, and automotive industries, where materials are subjected to complex loading conditions. The Al7075-T651 aluminum alloy is considered one of the strongest commercially available aluminum alloys, having a tensile strength comparable to that of many steels while maintaining a significantly lower weight. Flat plates manufactured from Al7075-T651 are widely used in structural applications requiring high strength, low weight, and high dimensional precision. These plates are employed in a broad range of engineering fields, including load-bearing aerospace structures (panels, ribs, spars, and frames), chassis components in transportation and automotive engineering, frames and structural elements for sports equipment, base plates and molds in the mechanical industry, as well as lightweight armored vehicle components and drone structures in the defense sector.

Cracks are among the most common causes of failure in engineering structures. Therefore, evaluating the safety and reliability of cracked mechanical components requires a thorough understanding of fracture mechanics. Fracture mechanics provides both theoretical and numerical tools for assessing the behavior of structures containing discontinuities and for predicting crack initiation and propagation under various loading conditions.

Due to the extensive use of aluminum alloy plates in engineering applications, numerous researchers have investigated their manufacturing, machining, and joining processes. For example, Magdalena Zawada-Michałowska and co-authors analyzed residual stresses as a function of milling conditions, coolant application, and cutting speed for the 2024-T351 aluminum alloy [1]. Their study highlighted the importance of determining both the type and magnitude of residual stresses, since these stresses are among the primary causes of post-machining deformation in thin-walled components [1].

Jun-Ren Zhao and collaborators investigated the effects of heat treatment and cold rolling on the mechanical properties of the Al6082 aluminum alloy. Their results showed that heat-treating the specimens exhibited improved elongation and higher strength. Furthermore, after cold rolling, both hardness and strength increased due to strain-hardening effects induced by plastic deformation [2].

Regarding the joining of aluminum alloy plates, Yanning Guo and Peiyao Li studied the influence of residual stresses and microstructure on fatigue crack propagation in welded joints made from 2024-T3 and 7075-T6 aluminum alloys. Their findings demonstrated that the presence of residual stresses accelerates fatigue crack growth [3].

Although Linear Elastic Fracture Mechanics (LEFM) and Failure Assessment Diagram (FAD) methodologies are well-established tools for structural integrity assessment, comparatively few studies have systematically investigated the influence of crack position and orientation on the structural integrity of Al7075-T651 plates subjected to tensile loading. Therefore, the objective of this study is to evaluate the effect of seven different crack configurations on stress intensity factors and structural integrity using finite element analysis and FAD assessment.

The main contribution of the current research consists in analyzing how crack geometry and orientation influence the Stress Intensity Factor (K) and how variations in crack position and orientation affect fracture risk according to the FAD approach. The proposed methodology combines numerical simulation with structural integrity assessment in order to provide a comprehensive evaluation of cracked thin plates subjected to uniaxial loading conditions. The results provide practical guidance for the inspection and integrity assessment of aluminum structural components containing crack-like defects.

### 1.1. Fundamentals of Fracture Mechanics

Cracks may develop in mechanical structural components during manufacturing and machining processes. In order to ensure the safety and reliability of these components, preventive measures must be implemented to detect cracks at an early stage and to prevent their propagation [4].

Fracture Mechanics is concerned with the manner in which material defects, particularly cracks, propagate under applied loading conditions. In the case of the Al7075-T651 aluminum alloy, this field is especially important because, despite its high mechanical strength, the material may exhibit brittle behavior and sensitivity to rapid crack propagation under certain conditions. The Stress Intensity Factor (K) is the most important parameter in fracture mechanics [5]. It characterizes the “stress state” at the crack tip when the component is subjected to linear-elastic loading conditions [6]. The value of K depends on the applied load, crack size, and the geometry of the component [7].

**Figure 1:**
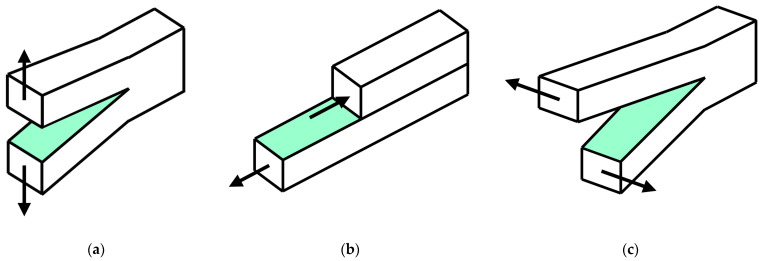
Crack propagation modes: (**a**) Mode I—Opening mode; (**b**) Mode II—Sliding mode; (**c**) Mode III—Tearing mode.

Crack propagation can occur according to the following three fundamental fracture modes [8]:

Mode I—Opening mode: The applied stress acts perpendicular to the crack plane, causing the crack faces to separate. This is the most common and the most critical fracture mode (Figure 1a).

Mode II—Sliding mode: In-plane shear stress acts perpendicular to the crack front, producing relative sliding between the crack surfaces (Figure 1b).

Mode III—Tearing mode: Shear stress acts parallel to both the crack plane and the crack front, leading to out-of-plane tearing deformation (Figure 1c).

All other crack propagation modes can be represented as combinations of these three fundamental modes. According to previous studies, the stress intensity factors K_I_ and K_II_ are the dominant parameters governing failure behavior under mixed-mode loading conditions [9].

The determination of the Mode I stress intensity factor, K_I_, remains an important topic in the analysis of cracked structures. Numerous researchers have evaluated stress intensity factors for different crack geometries in flat plates. For example, Cuesta I. I. and Alegre J. M. [10] determined the stress intensity factor for 15-5PH stainless steel using a Small Punch Test methodology, combining Failure Assessment Diagram analysis with numerical simulation of pre-cracked specimens [10].

Fayed A. S. investigated mixed-mode I and II stress intensity factors for plates containing inclined edge cracks [11]. In addition, Bhagat and co-authors studied and determined stress intensity factors for multiple inclined cracks subjected to biaxial tensile loading [12].

Brittle crack propagation occurs when the stress intensity factor *K* reaches a critical value, denoted by *K_Ic_*, known as the fracture toughness of the material. The fracture toughness *K_Ic_* is a material property and can be determined when plane strain conditions are satisfied, according to Equation (1):

(1) $$B,a\geq 2.5\cdot {\left(\frac{K_{Ic}}{\sigma_{y}}\right)}^{2}$$

where

$B\;\left[mm\right]$ is the plate thickness;

$a\;\left[mm\right]$ is the crack length;

$K_{Ic}\;\left[MPa\sqrt{m}\right]$ is fracture toughness;

$\sigma_{y}\;\left[MPa\right]$ is the yield strength of the material.

### 1.2. Structural Integrity Assessment Methods

The Failure Assessment Diagram (FAD) is an important tool used in structural integrity analysis, particularly for the assessment of components containing defects. The methodology is based on two parameters, considering separately the risks associated with fracture and plastic collapse. The diagram defines an acceptable operating region for defective components, ensuring that the structure does not fail either by fracture or by plastic deformation.

The FAD approach accounts for the influence of crack geometry, component configuration, loading conditions, and material properties. A component is considered unsafe when the assessment point reaches or exceeds the failure assessment curve defined by the FAD. Different forms of this curve can be generated for different levels of analysis depending on the available material properties [13].

The FAD methodology covers a wide range of material behavior, from brittle fracture under linear-elastic conditions to ductile overload under fully plastic conditions. The method is particularly suitable for welded structures because it can incorporate the effects of residual stresses. In addition, the FAD approach may also be applied to the analysis of ductile fracture [4].

In fracture mechanics, the FAD is a graphical tool used to evaluate the structural integrity of a component containing a crack-like defect. It allows the determination of whether a given combination of applied load and defect size is safe or likely to result in failure [14].

The FAD methodology replaces the conventional relationship between the three main fracture mechanics parameters, fracture toughness, defect size, and applied load, with a two-parameter representation [15]. This approach enables a two-dimensional assessment in which the coordinates are defined by the toughness ratio, *K_r_*, and the load ratio, L_r_.

Table 1 summarizes the classification of structural behavior according to the value of the load ratio *L_r_*.

By combining the toughness ratio *K_r_*, which characterizes brittle fracture, with the load ratio *L_r_*, which characterizes plastic collapse, the FAD methodology provides a unified structural integrity assessment covering the entire spectrum of failure mechanisms in metallic materials.

The equation describing the safety boundary curve in a FAD has evolved over time, from simple empirical expressions to more advanced formulations that incorporate the constitutive behavior of the material through the stress–strain relationship.

Modern structural integrity standards, such as R6 [16], BS 7910 [17], and API 579 [18], define several levels of assessment complexity. Among these, the most commonly used approach for routine engineering evaluations is the Level 2 assessment procedure, which provides a generalized solution based on an approximation of material behavior. This formulation is independent of the specific geometry of the component and is valid for *L_r_* ≤ *L_r_^max^* [4]:

(2) $$f\left(L_{r}\right)=\left(1-0.14\cdot L_{r}^{2}\right)\cdot \left(0.3+0.7\cdot e^{-0.65\cdot L_{r}^{6}}\right)$$

The mathematical relationship defined by Equation (2) presents the following characteristics:

- Elastic regime (*Lr* → 0): When the value of *Lr* is very small, the function *f*(*Lr*) approaches 1.0. In this case, failure is governed primarily by *K_I_*, according to Linear Elastic Fracture Mechanics (LEFM).
- Plastic collapse limit (*L_r_^max^*): The assessment curve terminates abruptly at a maximum value of *L_r_*. Beyond this limit, the component is considered to fail due to excessive plastic deformation, regardless of the crack size.

### 1.3. Finite Element Method

The Finite Element Method (FEM) is a numerical technique used to solve partial differential equations and represents an essential tool in engineering analysis and mathematical modeling. The finite element method subdivides a complex domain into smaller and simpler subdomains, called finite elements. This discretization process transforms differential equations into systems of algebraic equations that can be solved numerically. Each finite element is connected through points known as nodes, while the overall solution is obtained by combining the approximation functions associated with each element.

The FRANC2D version 4 software package [19] is specifically developed for the study of fatigue crack propagation and for the determination of the Stress Intensity Factor at the tip of crack-like geometric discontinuities, based on the results obtained from finite element analysis. The software was developed by a research group at Cornell University under the supervision of Anthony Ingraffea and Paul Wawrzynek [20].

The FRANC2D version 4 software package was selected because it is specifically designed for fracture mechanics applications and provides dedicated tools for crack analysis in two-dimensional structures [21]. Unlike general-purpose finite element software, FRANC2D automatically generates the crack tip singularity and performs adaptive local remeshing around the crack tip, ensuring an accurate representation of the stress field singularity and reliable computation of fracture mechanics parameters [22]. Furthermore, the software directly evaluates the stress intensity factors *K_I_* and *K_II_* based on Linear Elastic Fracture Mechanics (LEFM) principles and allows efficient modeling of crack propagation without requiring manual mesh reconstruction after each crack increment. These capabilities make FRANC2D particularly suitable for investigating the influence of crack position and orientation on the structural integrity of Al7075-T651 plates.

FRANC2D performs linear-elastic finite element analyses for structural components subjected to plane stress, plane strain, or axisymmetric conditions. The software also allows the definition of one or multiple cracks and enables the calculation of stress intensity factors associated with crack tip loading conditions [23].

The fracture analysis procedures implemented in FRANC2D are based on two-dimensional Linear Elastic Fracture Mechanics (LEFM) concepts. The stress intensity factors governing the fracture process within the LEFM framework are determined using displacement correlation techniques or modified crack closure methods.

When performing discrete crack analysis, not only is the crack geometry explicitly represented at each propagation step, but the mesh discretization must also be continuously updated to reflect the current crack configuration. The automatic remeshing strategy implemented in FRANC2D consists of deleting the finite elements surrounding the crack tip, advancing the crack tip position, and subsequently generating a trial discretization to connect the newly formed crack geometry with the existing mesh.

The determination of fracture mechanics parameters is carried out by defining one or more cracks within the analyzed structure. After crack definition, FRANC2D automatically removes the elements located near the crack tip and performs a local remeshing procedure that ensures the correct representation of the Stress and Strain Field Singularity. This crack-tip discretization may either be modified by the user or accepted in the automatically generated form provided by the software.

## 2. Geometry and Material Properties of the Plate

**Figure 2:**
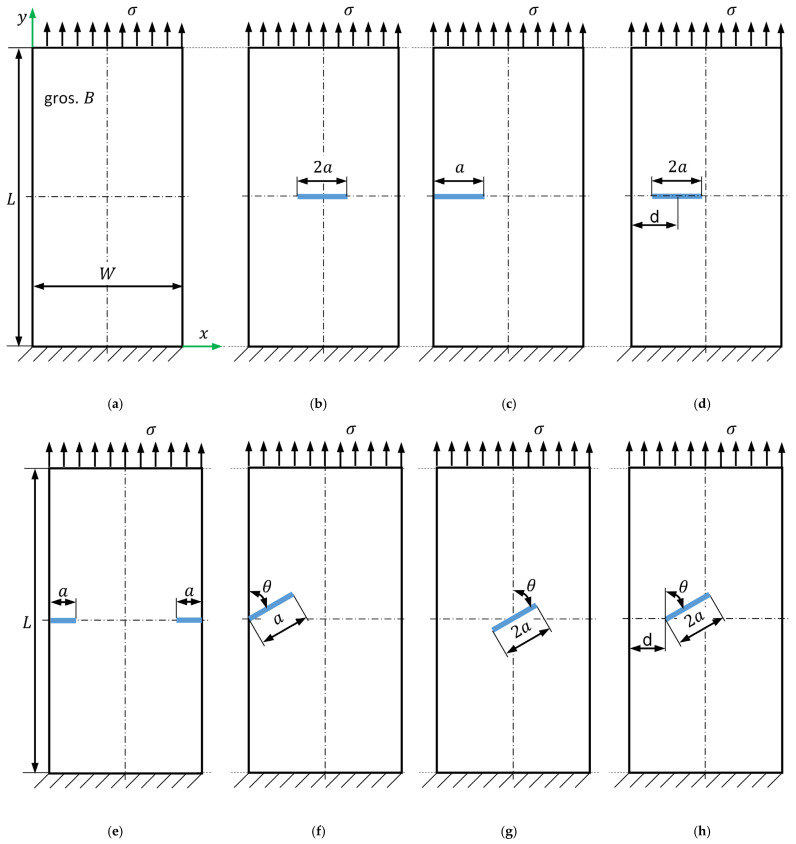
Analyzed Crack Configurations: (**a**) uncracked plate; (**b**) plate containing a central internal crack; (**c**) plate containing an edge crack; (**d**) plate containing an asymmetric internal crack; (**e**) plate containing two symmetric side cracks; (**f**) plate containing an inclined edge crack with θ = 60°; (**g**) plate containing an inclined central internal crack with θ = 60°; (**h**) plate containing an inclined asymmetric internal crack with θ = 60°.

The Finite Element Method analysis was performed on a flat plate with a length of *L* = 400 mm, a width of *W* = 100 mm, and a thickness of *B* = 10 mm, as illustrated in Figure 2a. The plate was clamped at one end and subjected to a uniaxial tensile stress of *σ* = 140 MPa at the free end.

The mechanical properties of the analyzed Al7075-T651 aluminum alloy are presented in Table 2.

The finite element analysis of the plate was carried out for cracks located at the mid-length section of the plate (*L* = 400 mm), according to the configurations presented in Figure 2:

(a) An uncracked plate;
(b) A plate containing a central internal crack with a total length of 2*a* = 12 mm positioned at the center of the plate, Figure 2b;
(c) A plate containing an edge crack with a length of *a* = 12 mm positioned at the mid-length of the plate, Figure 2c;
(d) A plate containing an asymmetric internal crack with a total length of 2*a* = 12 mm positioned at a distance of *d* = 10 mm from the left edge of the plate, Figure 2d;
(e) A plate containing two symmetric side cracks, each having a length of *a* = 6 mm and positioned at the mid-length of the plate, Figure 2e);
(f) A plate containing an inclined edge crack with a length of *a* = 12 mm and an inclination angle of *θ* = 60°, Figure 2f;
(g) A plate containing an inclined central internal crack with a total length of 2*a* = 12 mm and an inclination angle of *θ* = 60°, positioned at the center of the plate, Figure 2g;
(h) A plate containing an inclined asymmetric internal crack with a total length of 2*a* = 12 mm and an inclination angle of *θ* = 60°, positioned at a distance of *d* = 4 mm from the left edge of the plate (Figure 2h).

For all analyzed configurations, both the applied loading and the crack orientation were defined with respect to the Cartesian coordinate system (x, y) shown in Figure 2a.

The plate was analyzed under uniaxial tensile loading in order to isolate the influence of crack position and orientation on the stress intensity factors and structural integrity. Although engineering structures may be subjected to more complex loading conditions, such as bending, shear, or combined loading, the present study focuses on a fundamental loading case that allows a direct comparison between different crack configurations. All cracks were positioned at the mid-length of the plate (*L*/2 = 200 mm = 2*W*), corresponding to a distance equal to twice the plate width from the clamped end. At this location, the local stress perturbations induced by the clamped boundary condition are significantly attenuated, resulting in an approximately uniform stress field. Therefore, the influence of the stress concentration associated with the clamped end on the crack-tip stress field is considered negligible.

## 3. Results and Discussion

### 3.1. Analytical Calculation of Stress Intensity Factors

To validate the numerical results, the Stress Intensity Factor values obtained analytically were calculated and compared with those determined using the FRANC2D program. The stress intensity factor, *K_I_*, is calculated using the general relationship:

(3) $$K_{I}=\sigma \cdot \sqrt{\pi \cdot a}\cdot f\left(\frac{a}{W}\right)$$

where

$K_{I}\;\left[MPa\sqrt{m}\right]$ is the stress intensity factor;

$\sigma \;\left[MPa\right]$ is the applied stress acting on the plate;

$a\;\left[mm\right]$ is the crack length or half-crack length;

$f\left(\frac{a}{W}\right)\;\left[-\right]$ is the geometric correction factor, which depends on the crack geometry and crack position within the plate.

The calculated values of K_I_ obtained for the analyzed crack configurations are presented in Table 2 and Table 3.

The condition for brittle fracture propagation of a cracked component is defined by: $K_{I}\geq K_{Ic}$. If $K_{I}<K_{Ic}$ crack propagation does not occur. Here, *K_Ic_* represents the fracture toughness of the material.

For several crack configurations, analytical solutions for the stress intensity factors are available in the specialized fracture mechanics literature [24,25]. These include:

- Plate containing a central internal crack, shown in Figure 2b [24]:

(4) $$2a=12\;mm,\;2W=100\;mm\;\Rightarrow \frac{a}{W}=\frac{6}{50}=0.12\;f\left(\frac{a}{W}\right)=\frac{1-0.5\cdot \left(\frac{a}{W}\right)+0.370\cdot {\left(\frac{a}{W}\right)}^{2}-0.044\cdot {\left(\frac{a}{W}\right)}^{3}}{\sqrt{1-\frac{a}{W}}}=1.008\;K_{I}=\sigma \cdot \sqrt{\pi \cdot a}\cdot f\left(\frac{a}{W}\right)=140\cdot \sqrt{\pi \cdot 6}\cdot 1.008=612.69\;MPa\sqrt{mm}$$

- Plate containing an edge crack, as shown in Figure 2c [24]:

(5) $$a=12\;mm,\;W=100\;mm\;\Rightarrow \frac{a}{W}=\frac{6}{50}=0.12\;f\left(\frac{a}{W}\right)=1.122-0.231\cdot \left(\frac{a}{W}\right)+10.550\cdot {\left(\frac{a}{W}\right)}^{2}-21.710\cdot {\left(\frac{a}{W}\right)}^{3}+30.382\cdot {\left(\frac{a}{W}\right)}^{4}=1.215\;K_{I}=\sigma \cdot \sqrt{\pi \cdot a}\cdot f\left(\frac{a}{W}\right)=140\cdot \sqrt{\pi \cdot 12}\cdot 1.215=1044.41\;MPa\sqrt{mm}$$

- Plate containing two symmetric side cracks, Figure 2e [24]:

(6) $$a=6\;mm,\;W=50\;mm\;\Rightarrow \frac{a}{W}=\frac{6}{50}=0.12\;f\left(\frac{a}{W}\right)=\frac{1.122-0.561\cdot \left(\frac{a}{W}\right)-0.015\cdot {\left(\frac{a}{W}\right)}^{2}+0.091\cdot {\left(\frac{a}{W}\right)}^{3}}{\sqrt{1-\frac{a}{W}}}=1.124\;K_{I}=\sigma \cdot \sqrt{\pi \cdot a}\cdot f\left(\frac{a}{W}\right)=140\cdot \sqrt{\pi \cdot 6}\cdot 1.124=683.20\;MPa\sqrt{mm}$$

- A plate containing an inclined central internal crack with an inclination angle of θ = 60°, positioned at the center of the plate, shown in Figure 2g [25]:

(7) $$a=6\;mm,\;\beta =60{}^{\circ}\;K_{I}=\sigma \cdot \sqrt{\pi \cdot a}\cdot {\left(\sin\beta \right)}^{2}=140\cdot \sqrt{\pi \cdot 6}\cdot {\left(\sin60\right)}^{2}=455.87\;MPa\sqrt{mm}\;K_{II}=\sigma \cdot \sqrt{\pi \cdot a}\cdot \sin\beta \cdot \cos\beta =140\cdot \sqrt{\pi \cdot 6}\cdot \sin60\cdot \cos60=263.20\;MPa\sqrt{mm}$$

The analytical results obtained for the stress intensity factors were compared with the numerical results determined using the Finite Element Method implemented in FRANC2D. This comparison was performed in order to validate the correct implementation and application of the FRANC2D software version 4 in the analysis of cracked plate configurations.

### 3.2. Finite Element Modeling in FRANC2D

The geometry of the plate was generated using the Casca preprocessor, which was employed to create both the plate geometry and its finite element discretization. The plate contour was defined using the “Lines Connect” command. Subsequently, the nodal densities along the plate boundaries were specified using the “Subdivide” command.

The mesh discretization of the plate was then generated using Q8 and Bilinear4side quadrilateral finite elements. The final mesh consisted of approximately 10,000 nodes in order to achieve a sufficiently refined discretization, especially around the crack tip where high stress gradients occur. To ensure the accuracy of the finite element results, a sufficiently refined mesh was employed, with a characteristic element size of 2 mm. This mesh density is small compared with the crack length and provides stress intensity factor values that closely match the corresponding analytical solutions, as demonstrated by the low errors reported in Table 3. In addition, FRANC2D automatically performs local remeshing around the crack tip using significantly smaller elements, ensuring an accurate representation of the crack tip singularity and reliable computation of fracture mechanics parameters. Excessively coarse meshes may lead to inaccurate stress distributions and stress intensity factor values; therefore, the adopted mesh refinement strategy was considered appropriate for the present analysis. The geometry together with the generated mesh created in Casca was saved and subsequently imported into the FRANC2D finite element analysis program.

Within FRANC2D, the first step consisted of defining the type of analysis problem as a plane strain condition using the sequence PRE-PROCESS → PROBLEM TYPE → Plane Strain. The plane strain assumption is consistent with both the plate configuration and all analyzed crack geometries according to Equation (1). The validity of the plane strain assumption was verified using Equation (1). For the Al7075-T651 alloy, according to the values presented in Table 2, due to the fracture toughness being *K_Ic_* = 23 MPa√m and a yield strength being *σ_y_* = 500 MPa, the minimum thickness required to satisfy the plane strain condition is *B_min_* = 5.29 mm. Since the plate thickness used in the present study is B = 10 mm, the plane strain assumption is considered valid.

**Figure 3:**
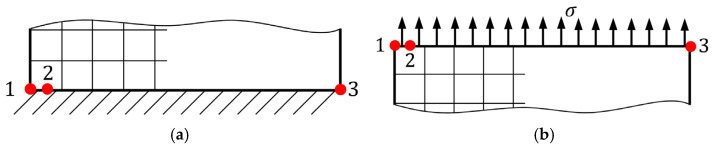
Boundary conditions and loading configuration of the plate: (**a**) boundary conditions; (**b**) loading configuration.

The next stage of the finite element analysis involved the specification of boundary conditions and the application of the tensile loading σ = 140 MPa, as follows:

- The lower edge of the plate was fully constrained using the command: PRE-PROCESS → FIXITY → FIX EDGE → xy, by specifying the start node (1), adjacent node (2), and end node (3), as shown in Figure 3a.
- The tensile load was applied along the upper edge of the plate using the command: PRE-PROCESS → LOADS → DIST LOAD → Y GLOBAL → CONSTANT, by specifying the start node (1), adjacent node (2), end node (3), and the stress value acting along the y-direction, σ = 140 MPa, as illustrated in Figure 3b.

After defining the geometry, material properties, boundary conditions, and loading, the stress and strain analysis of the plate could be performed. The calculation of stresses and displacements was carried out using the option: ANALYSIS → LINEAR → DIRECT STIF, through which the software solves the governing system of equations using the Gaussian elimination method.

The post-processing stage allows visualization of the finite element analysis results. The software provides graphical representations of the deformed plate configuration, the normal stress along the *y*-axis (SIG Y), the maximum principal stress (SIG 1), and the displacement field (DISP V). The results are displayed in the form of stress or deformation contour bands.

**Figure 4:**
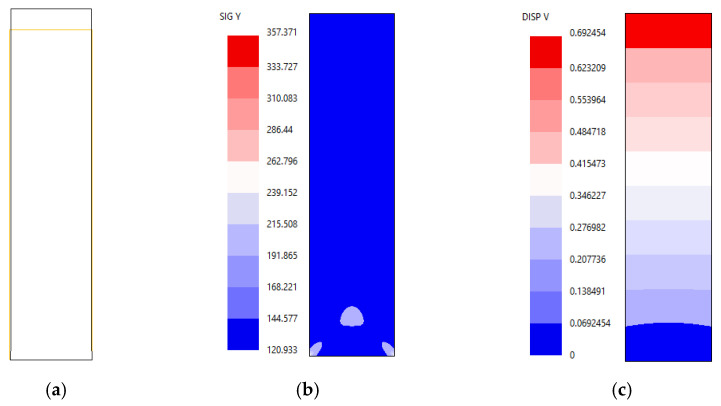
Finite element analysis results for the uncracked plate: (**a**) deformed shape of the plate; (**b**) normal stress SIG Y; and (**c**) displacement DISP V.

The results obtained for the uncracked plate are presented in Figure 4 as follows:

- the deformed shape of the plate is shown in Figure 4a, where it can be observed that no displacement occurs in the clamped region at the lower edge of the plate;
- the maximum normal stress occurs in the clamped region along the y-direction, with a value of SIG Y = 357.371 MPa, as shown in Figure 4b;
- the maximum displacement occurs along the y-direction and reaches a value of DISP V = 0.692 mm at the free end where the tensile load is applied, while in the clamped region the displacement is zero (DISP V = 0), as presented in Figure 4c.

For the determination of the Stress Intensity Factor values using FRANC2D, the corresponding crack geometries were defined for each analyzed case. For edge cracks, the crack initiation node and crack tip position were specified, whereas for internal cracks, the coordinates of the initial and final crack points were defined with respect to the Cartesian coordinate system (x, y) shown in Figure 2a.

**Figure 5:**
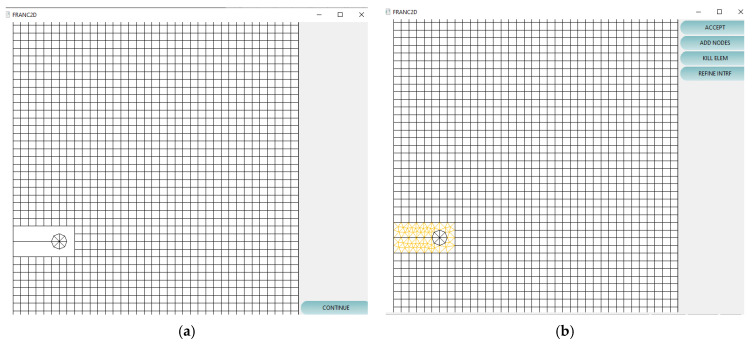
Crack generation in FRANC2D (edge crack case). (**a**) Generation of the crack tip singularity. (**b**) Remeshing around the crack tip (highlighted with yellow).

FRANC2D automatically generates the Crack Tip Singularity at the crack tip, as illustrated in Figure 5a, and also performs automatic remeshing in the crack-tip region, as shown in Figure 5b.

**Figure 6:**
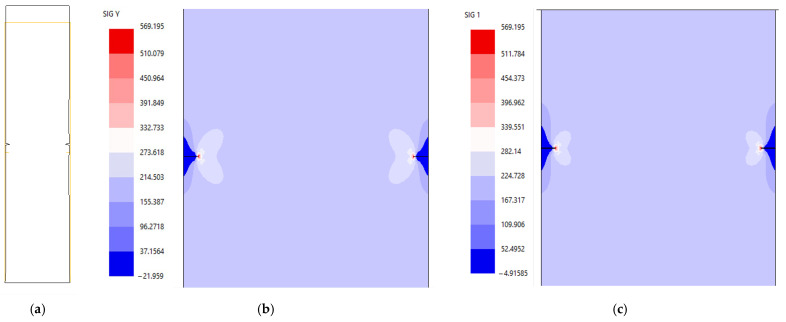
FRANC2D simulation results (plate containing two symmetric side cracks): (**a**) deformed shape of the plate; (**b**) normal stress SIG Y; and (**c**) principal stress SIG 1.

After running the simulation and solving the governing equations using the Gaussian elimination method, the software provides the deformed configuration of the cracked plate, the normal stress distribution SIG Y along the y-direction, and the maximum principal stress SIG 1, which indicates the preferred crack propagation direction, as illustrated in Figure 6.

Analysis of Figure 6 shows that the deformation of the plate occurs primarily along the *y*-axis, while the displacements along the *x*-axis remain negligible. Furthermore, the normal stress SIG Y and the principal stress SIG 1 reach the same maximum value at the crack tip: SIG Y = SIG 1 = 569.2 MPa. For all analyzed crack configurations, the maximum normal and principal stresses occur at the crack tip.

**Figure 7:**
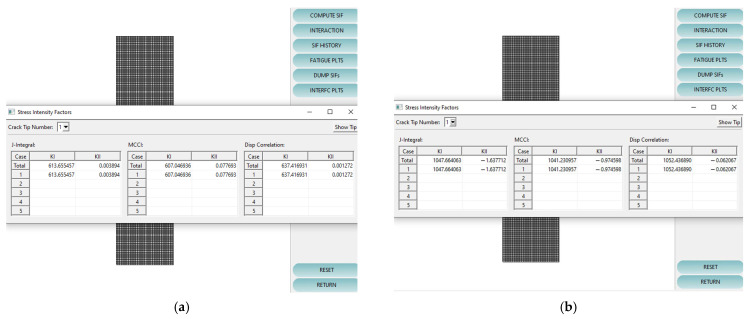
Stress intensity factors generated by FRANC2D: (**a**) Central internal crack; (**b**) Edge crack.

In addition, the finite element analysis performed with FRANC2D provided the stress intensity factors corresponding to each crack configuration. For straight internal and edge cracks, the dominant parameter is the Mode I stress intensity factor associated with the opening mode of crack propagation, as shown in Figure 7.

**Figure 8:**
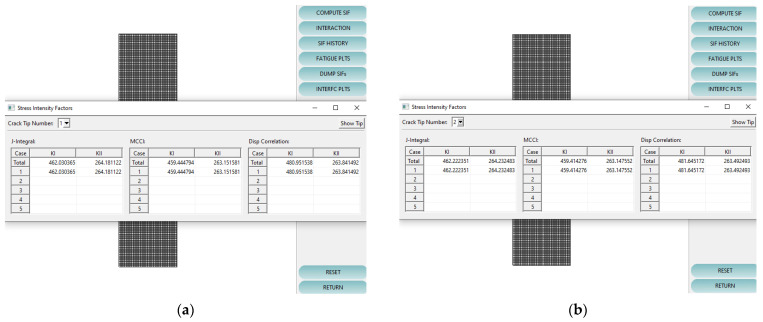
Stress intensity factors for an inclined crack located at the center of the plate: (**a**) Initial crack tip; (**b**) Final crack tip.

For straight cracks, the results indicate the dominant contribution of the Mode I stress intensity factor *K_I_* compared with the Mode II stress intensity factor *K_II_*, which corresponds to the sliding mode of crack propagation. For internal or inclined cracks, both *K_I_* and *K_II_* stress intensity factors were obtained at the initial and final crack tips, as illustrated in Figure 8.

The results obtained from the finite element analysis using FRANC2D for all seven cracked plate configurations are summarized in Table 3.

Table 4 presents the errors between the analytical solutions available in the literature for the corresponding crack geometries and orientations and the numerical solutions obtained using the Finite Element Method implemented in FRANC2D. The error was calculated using the following relationship:

(8) $${∆}_{\left(K_{I}\right)}=\frac{K_{I}^{An}-K_{I}^{Franc2D}}{K_{I}^{An}}\cdot 100$$

The analysis of Table 4 indicates very small differences between the analytical calculations and the finite element results obtained using FRANC2D. These results confirm the accuracy and correct implementation of the finite element analysis methodology used in the present study.

### 3.3. Failure Assessment Diagram (FAD) Analysis

The Failure Assessment Diagram is based on two principal structural failure criteria: final plastic collapse and Linear Elastic Fracture Mechanics (LEFM). Plastic collapse occurs when a substantial portion of the structure undergoes significant plastic deformation, leading to unstable structural behavior. In this situation, failure is governed primarily by excessive plastic deformation rather than by crack propagation.

The FAD methodology enables the assessment of structural safety using two nondimensional parameters:

The load ratio *L_r_* measures the proximity of the structure to plastic collapse and is defined as:

(9) $$L_{r}=\frac{\sigma_{ef}}{\sigma_{y}}$$

where *σ_ef_* is the effective stress in the plate and *σ_y_* is the yield strength of the plate material.

For the cracked plate configurations analyzed in this study, the effective stress *σ_ef_* is calculated using the following relationship:

(10) $$\sigma_{ef}=\sigma \cdot \frac{W}{W-a_{ef}}$$

where *a_ef_* = *a* for straight cracks and *a_ef_* = *a·sinθ* for inclined cracks.

The fracture ratio *K_r_* measures the proximity of the structure to brittle fracture propagation and is defined as:

(11) $$K_{r}=\frac{K_{ef}}{K_{Ic}}$$

where *K_ef_* is the effective stress intensity factor and *K_Ic_* is the fracture toughness of the plate material.

The FAD also includes a limiting value *L_r_^max^*, defined as [10]:

(12) $$L_{r}^{max}=\frac{\sigma_{y}+\sigma_{u}}{2\cdot \sigma_{u}}$$

where *σ_y_* is the yield strength and *σ_u_* is the ultimate tensile strength (UTS) of the material.

For the analyzed Al7075-T651 aluminum alloy, the yield strength is *σ_y_* = 500 MPa and the ultimate tensile strength is *σ_u_* = 550 MPa (Table 2), leading to a maximum value of:

(13) $$L_{r}^{max}=\frac{\sigma_{y}+\sigma_{u}}{2\cdot \sigma_{y}}=\frac{500+550}{2\cdot 500}=1.05$$

The boundary curve of the FAD is defined by Equation (2). A structure is considered safe when the assessment point corresponding to a given crack configuration (*L_r_*, *K_r_*) lies below the FAD curve. If the point lies directly on the curve, the structure is considered to be at the failure limit, whereas points located above the curve indicate structural failure.

Based on these considerations, the effective crack length *a_ef_*, effective stress *σ_ef_*, and load ratio *L_r_* corresponding to the analyzed crack configurations are presented in Table 5.

Inclined cracks introduce mixed-mode loading conditions, increasing the risk of unstable crack propagation in the cracked plate. For inclined cracks, the effective stress intensity factor is determined using the relationship:

(14) $$K_{ef}=\sqrt{K_{I}^{2}+K_{II}^{2}}$$

The calculated values of the effective stress intensity factor and the fracture ratio for all analyzed crack configurations are presented in Table 6.

In order to evaluate the safety of each cracked plate configuration, Table 7 presents the coordinates of the assessment points (*L_r_*, *K_r_*) corresponding to each analyzed crack type.

**Figure 9:**
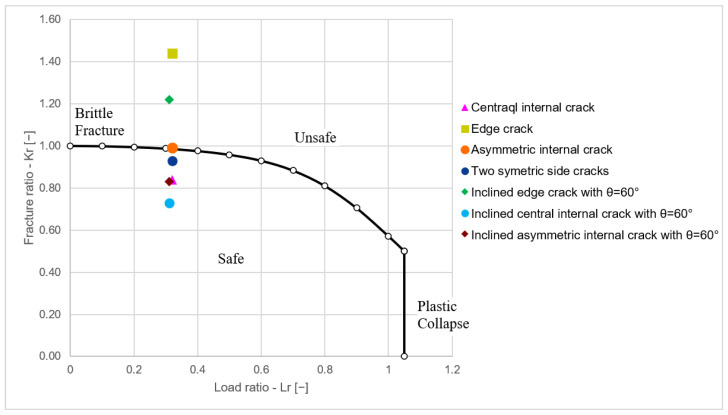
FAD for the analyzed crack configurations.

Based on the data presented in Table 7, the position of each assessment point corresponding to the analyzed crack configurations was plotted on the FAD, as shown in Figure 9.

The analysis of the FAD leads to the following observations:

- The plate containing a straight edge crack (Figure 2c) and the plate containing an inclined edge crack (Figure 2f) exhibit low values of *L_r_*, indicating the absence of generalized plastic yielding. However, both configurations fail by brittle fracture propagation due to the high stress concentration at the crack tip. These two crack configurations generate the highest fracture ratio values, exceeding the critical limit *K_r_* = 1.
- The plate containing an asymmetric internal crack (Figure 2d) is close to the failure limit, with a fracture ratio *K_r_* approaching the critical value *K_r_* = 1.
- The plate containing two symmetric side cracks (Figure 2e) also approaches the structural failure limit.
- The plate containing a central internal crack (Figure 2b), the plate containing an inclined central internal crack (Figure 2g), and the plate containing an inclined asymmetric internal crack (Figure 2h) remain within the safe region of the FAD.

## 4. Conclusions

The analysis of the Stress Intensity Factor together with the Failure Assessment Diagram enables the evaluation of the influence of crack position on the structural integrity of an aluminum plate. The main conclusions derived from the present study are summarized as follows:

1. All assessment points are vertically aligned on the FAD because the applied loading acting on the plate remains constant for all analyzed cases. Consequently, the observed differences are governed primarily by crack geometry and crack position.
2. For certain crack configurations (cases d, f, and h), analytical calculations become less accurate. In the case of inclined cracks, in addition to the Mode I stress intensity factor *K*_I_, the Mode II component *K_II_* associated with shear loading must also be considered. The FRANC2D program provides significantly more accurate simultaneous evaluations of both *K_I_* and *K_II_*.
3. In the case of plates containing two symmetric side cracks (case e), the stress fields associated with each crack may interact when the cracks are sufficiently close to one another. The Finite Element Method captures this interaction more accurately than standard analytical relationships. The results indicate that two edge cracks of 6 mm each are less critical than a single edge crack of 12 mm due to the redistribution of the stress flow within the plate.
4. All assessment points are grouped along the *L_r_* axis within the range of approximately 0.31–0.32. This indicates that the structure remains far from generalized plastic yielding because the applied stress of 140 MPa is relatively low compared with the material yield strength (*σ_y_* = 500 MPa).
5. The FAD analysis shows that, for the analyzed Al7075-T651 aluminum alloy, most assessment points are positioned relatively high along the vertical axis corresponding to *K_r_*. This behavior confirms that the material tends to fail predominantly through brittle fracture propagation before significant plastic deformation develops. None of the analyzed cases exceed *L_r_* = 0.5, which indicates that all configurations remain far from the plastic collapse limit corresponding to *L_r_* = 1.0.
6. Using FRANC2D, crack propagation can also be simulated numerically. Although the present study focuses primarily on the influence of crack position within the plate, the finite element analysis performed with FRANC2D additionally allows the prediction of the fatigue crack propagation direction according to the direction of the maximum principal stress (SIG 1), based on the maximum circumferential stress criterion.
7. The results obtained demonstrate that crack position has a decisive influence on fracture behavior. Edge cracks represent the most critical crack configuration. A crack having the same total length becomes significantly more dangerous when located at the plate edge (Figure 2c) compared with a centrally located internal crack (Figure 2b).
8. Furthermore, the plate containing an asymmetric internal crack (Figure 2d) and the plate containing two symmetric side cracks (Figure 2e) approach the structural failure limit and therefore require periodic inspection and structural monitoring during service operation.
9. The present study is limited to uniaxial tensile loading. Future investigations will consider more complex loading conditions, including bending, shear, and combined loading, in order to further evaluate the influence of crack position and orientation on the structural integrity of Al7075-T651 plates.

## Figures and Tables

**Table 1:** Structural behavior as a function of the load ratio *L_r_.*

Load Ratio (*L_r_*) [−]	Mechanical Properties
*L_r_* < 1	The applied stress remains below the yield strength of the material. The structural response is predominantly elastic, and the plastic zone at the crack tip is negligible.
*L_r_* = 1	The net-section stress reaches the yield strength of the material.
*L_r_* > 1	The structure enters the generalized plastic yielding regime.

**Table 2:** Mechanical properties of the Al7075-T651 aluminum alloy.

Material	Mechanical Properties				
	Modulus of Elasticity $E\;\left[MPa\right]$	Yield Strength $\sigma_{y}\;[MPa]$	Ultimate Tensile Strength $\sigma_{u}\;[MPa]$	Poisson’s Ratio $\nu \;\left[-\right]$	Fracture Toughness K_Ic_ [$MPa\sqrt{m}]$
Al7075-T651	71,500	500	550	0.33	23

**Table 3:** *K_I_* and *K_II_* Results Obtained Using FRANC2D.

Crack Configuration	$K_{I}\;\left[MPa\sqrt{mm}\right]$	$K_{II}\;\left[MPa\sqrt{mm}\right]$
Central internal crack, Figure 2b	613.66	0
Edge crack, Figure 2c	1047.66	0
Asymmetric internal crack, Figure 2d	719.89	0
Two symmetric side cracks, Figure 2e	679.00	0
Inclined edge crack with θ = 60°, Figure 2f	845.07	279.38
Inclined central internal crack with θ = 60°, Figure 2g	462.22	264.23
Inclined asymmetric internal crack with θ = 60°, Figure 2h	526.74	294.65

**Table 4:** Error between the analytical calculation and the FRANC2D results for *K_I_*.

Crack Configuration	$K_{I}^{An}\;\left[MPa\sqrt{m}\right]$	$K_{I}^{Franc2D}\;\left[MPa\sqrt{m}\right]$	Error [%]
Central internal crack, Figure 2b	612.69	613.66	−0.16
Edge crack, Figure 2c	1044.41	1047.66	−0.31
Two symmetric side cracks, Figure 2e	683.2	679.00	0.61
Inclined central internal crack with θ = 60°, Figure 2g	455.87	462.22	−1.39

**Table 5:** Effective stress and load ratio results.

Crack Configuration	$a_{ef}\;\left[mm\right]$	$\sigma_{ef}\;\left[MPa\right]$	$L_{r}\;[-]$
Central internal crack, Figure 2b	12	159.10	0.32
Edge crack, Figure 2c	12	159.10	0.32
Asymmetric internal crack, Figure 2d	12	159.10	0.32
Two symmetric side cracks, Figure 2e	12	159.10	0.32
Inclined edge crack with θ = 60°, Figure 2f	10.4	156.25	0.31
Inclined central internal crack with θ = 60°, Figure 2g	10.4	156.25	0.31
Inclined asymmetric internal crack with θ = 60°, Figure 2h	10.4	156.25	0.31

**Table 6:** Effective stress intensity factor and fracture ratio results.

Crack Configuration	$K_{ef}\;\left[MPa\sqrt{m}\right]$	$K_{r}\;[-]$
Central internal crack, Figure 2b	613.66	0.84
Edge crack, Figure 2c	1047.66	1.44
Asymmetric internal crack, Figure 2d	719.89	0.99
Two symmetric side cracks, Figure 2e	679.00	0.93
Inclined edge crack with θ = 60°, Figure 2f	890.00	1.22
Inclined central internal crack with θ = 60°, Figure 2g	532.41	0.73
Inclined asymmetric internal crack with θ = 60°, Figure 2h	603.55	0.83

**Table 7:** Position of the assessment points according to crack configuration.

Crack Configuration	$L_{r}\;[-]$	$K_{r}\;[-]$
Central internal crack, Figure 2b	0.32	0.84
Edge crack, Figure 2c	0.32	1.44
Asymmetric internal crack, Figure 2d	0.32	0.99
Two symmetric side cracks, Figure 2e	0.32	0.93
Inclined edge crack with θ = 60°, Figure 2f	0.31	1.22
Inclined central internal crack with θ = 60°, Figure 2g	0.31	0.73
Inclined asymmetric internal crack with θ = 60°, Figure 2h	0.31	0.83

## Data Availability

The original contributions presented in this study are included in the article. Further inquiries can be directed to the corresponding author.
